# Low-Histamine Diets: Is the Exclusion of Foods Justified by Their Histamine Content?

**DOI:** 10.3390/nu13051395

**Published:** 2021-04-21

**Authors:** Sònia Sánchez-Pérez, Oriol Comas-Basté, M. Teresa Veciana-Nogués, M. Luz Latorre-Moratalla, M. Carmen Vidal-Carou

**Affiliations:** 1 Departament de Nutrició, Ciències de l’Alimentació i Gastronomia, Facultat de Farmàcia i Ciències de l’Alimentació, Campus de l’Alimentació de Torribera, Universitat de Barcelona (UB), Av. Prat de la Riba 171, 8921 Santa Coloma de Gramenet, Spain; soniasanchezperez@ub.edu (S.S.-P.); oriolcomas@ub.edu (O.C.-B.); veciana@ub.edu (M.T.V.-N.); mariluzlatorre@ub.edu (M.L.L.-M.); 2 Institut de Recerca en Nutrició i Seguretat Alimentària (INSA·UB), Universitat de Barcelona (UB), Av. Prat de la Riba 171, 8921 Santa Coloma de Gramenet, Spain; 3 Xarxa d’Innovació Alimentària (XIA), C/Baldiri Reixac 4, 8028 Barcelona, Spain

**Keywords:** histamine, low-histamine diet, histamine-free diet, histamine intolerance, biogenic amines, histamine-releasing foods

## Abstract

A low-histamine diet is currently the most advised strategy to prevent the symptomatology of histamine intolerance. Conceptually, these diets should be founded on the exclusion of histamine-containing foods, although a certain disparity is found within the list of excluded foods in accordance with the different low-histamine diets available in the literature. This study aimed to critically review low-histamine diets reported in the scientific literature, according to the histamine and other biogenic amine contents of the excluded foods. A total of ten scientific studies that provided specific recommendations on the foods that must be avoided within the framework of a low-histamine diet were found. Overall, the comparative review brought out the great heterogenicity in the type of foods that are advised against for histamine intolerant individuals. Excluded foods were, in most cases, different depending on the considered diet. Only fermented foods were unanimously excluded. The exclusion of 32% of foods could be explained by the occurrence of high contents of histamine. The presence of putrescine, which may interfere with histamine degradation by the DAO enzyme at the intestinal level, could partly explain the reason why certain foods (i.e., citrus fruits and bananas) were also frequently reported in low-histamine diets. Finally, there was a range of excluded foods with an absence or very low levels of biogenic amines. In this case, certain foods have been tagged as histamine-liberators, although the mechanism responsible has not yet been elucidated.

## 1. Introduction

In recent years, a significant increase in the frequency of food intolerances (i.e., non-toxic and non-immune mediated reactions to food) has been detected in most developed societies. Food intolerances are disabling disorders that provoke an important decrease in the quality of life of this population [1,2]. Among them, histamine intolerance, also referred to as food histaminosis or hypersensitivity to food histamine, arises from the failure of the diamine oxidase (DAO) enzyme to degrade dietary histamine at the intestinal level. DAO deficit results in an increase in systemic histamine concentrations and the subsequent onset of symptoms [3,4,5]. This enzyme deficiency may have a genetic or pathological etiology (i.e., secondary to certain inflammatory bowel diseases) [6,7]. Moreover, some widely used pharmacologic drugs have also been described as potential DAO inhibitors, although in a punctual and reversible manner [8,9]. More recently, a potential etiological relationship has been suggested between a dysbiosis in the intestinal microbiota and histamine intolerance, although this hypothesis still needs to be further studied [10].

Plasma histamine accumulation can provoke a wide number of nonspecific gastrointestinal and extraintestinal clinical manifestations (i.e., dermatological, respiratory, neurological and hemodynamic complaints) [3,4,5]. The most frequent and severe symptoms, according to a recent comprehensive study, were abdominal distension, diarrhea, postprandial fullness, abdominal pain and constipation, followed by headaches, dizziness, and palpitations [2]. Moreover, in 97% of cases the onset of three or more symptoms concerning different organs were reported, thus, underlining the complexity of the clinical picture of histamine intolerance [2].

Currently, the most advised strategies to prevent the onset of symptoms are the follow-up of a low-histamine diet and the supplementation with exogenous DAO enzyme to enhance the intestinal histamine degradation [11,12,13]. As regards low-histamine diets, several clinical studies are continuously gathering increasing evidence on their efficacy on the improvement or remissions of symptoms [5,14,15,16,17,18,19,20,21,22,23]. The vast majority of these studies report efficacy rates higher than 70%, although some of them face certain limitations in terms of the number of patients and/or in the duration of the dietary intervention [3].

Conceptually, low-histamine diets should be based on the exclusion of histamine-containing foods. Histamine in foods is mainly formed by the bacterial decarboxylation of its precursor amino acid, histidine [24]. Therefore, foods susceptible to accumulating high contents of this amine are those that are microbiologically altered by spoilage bacteria; fermented products, due to the histaminogenic capacity of fermentative bacteria [25].

The design of a low-histamine diet is challenging due to different handicaps. One of these is the lack of consensus on the histamine level below which a food is said to be considered low in histamine. Thus, variable histamine levels in food ranging between 5–50 mg/kg have been pointed out as potential thresholds, while other authors are much more demanding and consider foods with low histamine concentrations to be those that contain amounts below 1 mg/kg [12,26,27,28]. Moreover, there is no specific regulation for the food industry to declare the occurrence or absence of histamine in food labelling, which could help histamine intolerant individuals to make suitable and informed choices.

Overall, providing dietary recommendations and guidelines in the frame of a low-histamine diet is difficult for healthcare professionals. In fact, disparity is found in the list of excluded foods reported by the different available low-histamine diets. Therefore, the aim of this work was to critically review the low-histamine diets from the literature, according to the contents of histamine and other biogenic amines found in the excluded foods.

## 2. Materials and Methods

### 2.1. Identification of Low-Histamine Diets

A selective search of scientific articles concerning low-histamine diets was performed through the PubMed and Web of Science bibliographic databases using the following keywords: “low-histamine diet”, “histamine-free diet”, ”histamine elimination diet”, “histamine restricted diet”, “histamine intolerance treatment” and “dietary management of histamine intolerance”. Only studies that clearly specified the foods allowed and/or excluded within a low-histamine diet were considered for further assessment.

### 2.2. Content of Histamine and Other Biogenic Amines in Foods and Beverages Excluded from Low-Histamine Diets 

Data on histamine and other biogenic amines (i.e., putrescine, cadaverine, tyramine, spermidine and spermine) content in foods and beverages from the Spanish market were obtained from the self-produced and updated database [11,29].

## 3. Results

**Figure 1:**
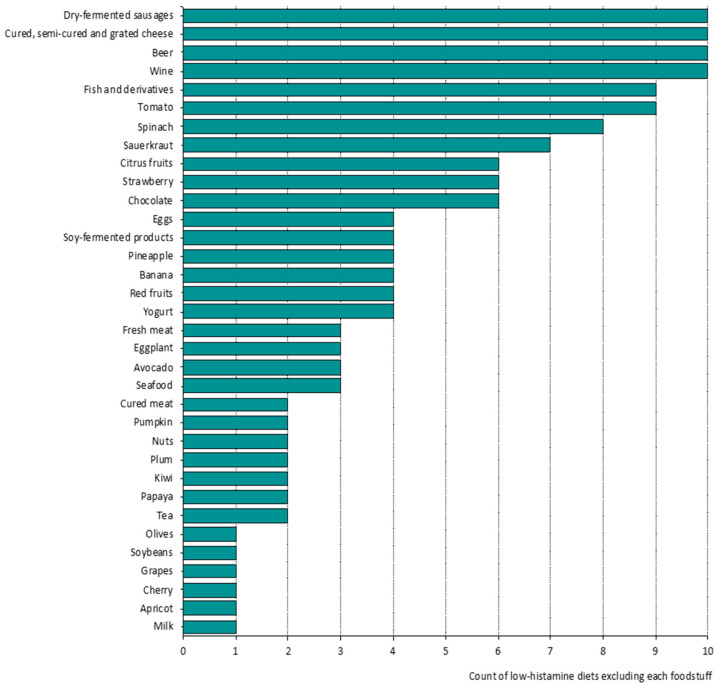
List of the avoided foods according to the literature review on low-histamine diets and count of references that exclude each foodstuff [14,16,17,20,23,26,30,31,32,33].

The selective search performed in this study resulted in a total of ten scientific publications, which provided specific recommendations about the range of foods that may be consumed or must be avoided within the framework of a low-histamine diet [14,16,17,20,23,26,30,31,32,33]. Overall, most of these studies based their recommendations on previous studies (mainly dealing with histamine contents in foods) or on those foods that patients associated with the onset of symptoms. Figure 1 graphically summarizes the extensive list of the avoided foods according to the literature review and the count of low-histamine diets that exclude each foodstuff. This comparative review showed great heterogenicity in the foods that should be excluded by histamine intolerant people. Firstly, all low-histamine diets unanimously advised the elimination of many fermented foods and beverages (i.e., dry-fermented sausages, cured cheese, wine and beer). On the other hand, it is worth highlighting that the majority of foods were only excluded by 50% or less of the revised diets. These results confirm the lack of consensus that currently exists in this type of diet.

**Figure 2:**
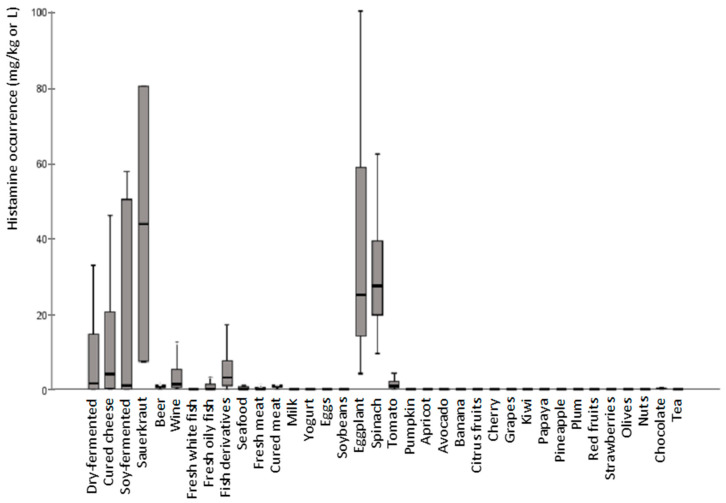
Histamine distribution (mg/kg or L) in foods marketed in Spain excluded from low-histamine diets [11,29].

The distribution of histamine levels in all excluded foods is shown in Figure 2. The exclusion of all foods listed above could not be explained by the occurrence of histamine. In fact, most of the foodstuffs retailed in Spain showed histamine levels below 1 mg/kg, considered by some authors as the threshold to define a food low in histamine. Contrarily, fermented foods had a large variability, even within samples of the same production batch due to the essentially microbial origin of histamine. Moreover, the nature of the food, the bacterial strain and many other factors that influence the growth and metabolic activity of the bacteria can also have an impact on the accumulation of this compound. This variability is precisely one of the causes of the complexity of issuing recommendations and, consequently, as a precautionary measure all the foods, that a priori may contain histamine, are eliminated.

Fermented foods (i.e., dry-fermented sausages, cured-cheese, sauerkraut and soy-fermented derivatives) are products that can potentially accumulate high histamine contents (Figure 2). In fermented foods, the presence of histamine depends on both the hygienic quality of the raw materials and/or manufacturing processes, and the histaminogenic capacity of technological bacteria [34,35]. As can be seen in Table 1, histamine levels in fermented foods marketed in Spain were relatively low (with mean values ranging between 22 and 74 mg/kg), but in certain cases these types of foods reached high histamine levels, with 5% of samples measuring above 203 mg/kg in cheese, 130 mg/kg in dry-fermented sausages and 486 mg/kg in soy-fermented products. Apart from histamine, in this food category, other biogenic amines could be frequently found, mainly tyramine. The presence of tyramine is strongly associated with the enzymatic activity of many fermentative lactic acid bacteria species. Maximum tyramine levels of 750 mg/kg in dry-fermented products, 1500 mg/kg in cheese and 1700 mg/kg in fermented vegetables were found. The occurrence of putrescine and cadaverine was also frequent, though at lower and more variable levels than tyramine (Table 1).

In fermented beverages (e.g., wine and beer) histamine and other biogenic amine contents were much lower than those reported for other fermented foods. However, it must be noted that the presence of alcohol will enhance the toxic effect of histamine [11,36,37]. In fact, alcohol and its metabolite, acetaldehyde, compete with histamine for the enzyme responsible for their metabolization (aldehyde dehydrogenase), thus, resulting in the accumulation of this amine in the organism [35,38].

Fish and fish derivatives are also usually excluded in low-histamine diets. As shown in Table 1, in most of the fresh fish and derivatives retailed in Spain (i.e., semi-preserved and preserved) no histamines or only in low amounts were detected (P95 below 20 mg/kg). The low occurrence of histamine was also reported by EFSA for this same food category. In fact, only 27% of a total of 6329 European fishery products showed histamine, and generally at low levels [38]. Nevertheless, high histamine concentrations could be achieved in the case of inadequate freshness of raw fish and/or hygienic deficiencies during the manufacturing of fish derivatives. An example is the 111 mg/kg found in fresh salmon and the 657 mg/kg of histamine determined in canned sardines (Table 1). For this reason, low histamine diets usually advise the against the consumption of fish, specifically certain scombroid species (e.g., mackerel, tuna, sardines and anchovy), which are susceptible to histamine accumulation due to their high free histidine contents [25]. Moreover, the action of some of spoilage bacteria derived from the lack of freshness could also entail the formation of other amines, especially putrescine and cadaverine (Table 1). The thermostable nature of biogenic amines implies that thermal treatments applied for the obtention of preserved canned fish do not help diminish their occurrence [5].

As regards fresh, cooked and cured meat, as in fish, the absence of histamine or other biogenic amines is expected, so long as freshness and correct hygienic conditions of the products or manufacturing processes are guaranteed (Table 1). In fact, as can be seen in Figure 1, fresh and cured meat were only excluded by three and two low-histamine diets, respectively. However, the presence of these foods in most low-histamine diets could entail a risk for histamine intolerant individuals if freshness is not guaranteed.

Histamine was also found in some plant-origin products, such as tomato, eggplant and spinach (Figure 2). The origin of low histamine levels in these foods may be physiological, but a high accumulation of this amine has been related to bacterial decarboxylase activity which occurs during storage [11,39]. The study performed by Lavizzari et al., showed a significant increase in histamine concentrations in spinach samples during 15 days of refrigerated storage [39]. The relatively high pH of spinach would allow for the implantation and growth of some Gram-negative bacteria (Enterobacteriaceae and Pseudomonadaceae groups), which would ultimately be responsible of histamine formation [39].

On the other hand, 68% of the not-allowed foods in low-histamine diets did not show significant histamine levels in any of the analyzed samples (Figure 2). Among them, citrus fruits, bananas, soybeans, pumpkins and nuts showed relevant amounts of putrescine (Table 1). In the case of citrus fruits, outstanding levels of putrescine are very often found, with mean levels of 79 mg/kg and maximum levels up to 173 mg/kg in the case of mandarines. In fact, despite the absence of histamine in citrus fruits, 60% of the revised low-histamine diets advised for their exclusion (Figure 1). Paradoxically, there are also certain foods with relevant amounts of putrescine, the exclusion of which is not listed in low-histamine diets [11,36,37]. This is the case for zucchini, peas, green peppers and sweet corn, among others, which could achieve maximum levels of putrescine ranging 25–150 mg/kg, depending on the product.

It has been reported that certain biogenic amines, mainly putrescine and cadaverine, could interfere with histamine degradation by the DAO enzyme at the intestinal level, being responsible for the major absorption and subsequent toxic potential enhancement of histamine. However, there is still scarce experimental evidence supporting this working hypothesis. The studies carried out years ago by Arunlakshana et al. (1954), Mongar (1957) and Hui and Taylor (1985) pointed out this potentially inhibitory effect of other biogenic amines on histamine metabolism both through in vitro assays and in animal models [40,41,42]. Concretely, Mongar (1957) observed that different aliphatic diamines, such as putrescine and cadaverine, could potentiate histamine-induced contractions of guinea pig ileum due to the fact that they could competitively inhibit the DAO enzyme [41].

Apart from the foods containing histamine or significant levels of putrescine, there was a wide range (53%) of excluded foods without histamine and with no detected or a low/very low occurrence of putrescine, cadaverine, tyramine, spermidine and/or spermine. Thus far, the available evidence does not help to explain to what extent the presence of low levels of these amines (alone or combined) may interfere with histamine degradation by the DAO enzyme and be responsible for the triggering of the symptoms of histamine intolerance. According to our knowledge, there is only one study on this topic, which was performed back in 1985 by Hui and Taylor, demonstrating the inhibitory effect of putrescine and cadaverine on histamine degradation in rats when these amines were present at levels four to five-fold higher [42]. Curiously, as may be seen in Figure 1, the vast majority of these foods are only excluded in one to three of the low-histamine diets, with the exception of chocolate, strawberry, eggs, pineapple and yogurt.

Moreover, certain foods have been tagged as histamine-liberators, as they could trigger the release of endogenous histamine. The list of foods with suggested histamine-releasing capacity, that may be found in various scientific articles, includes citrus fruits, seafood, papaya, tomato, nuts, pineapple, spinach, chocolate and strawberries, among others [4,5,43]. However, the mechanism responsible for this potential effect has not yet been elucidated. In fact, the only available extensive review as regards the putative histamine-releasing capacity of certain foods performed by Vlieg-Boerstra et al. (2005), clearly stated that there is a lack of evidence supporting this mechanism. There are no clinical studies in humans supporting the widely held belief that foods could have the ability to release histamine and this hypothesis is only based on few and no conclusive in vitro or animal studies [43].

In summary, while the evidence supporting the clinical efficacy of low-histamine diets is progressively growing, there is still a lack of consensus on the foods that must be avoided in the dietary management of histamine intolerance. The critical review performed herein demonstrates that the exclusion of only 32% of foods could be justified by their histamine content. The presence of other biogenic amines could partly help to explain the relationship that some patients have established between the consumption of certain histamine-free foods and the onset of symptoms. However, low-histamine diets continue to require the attention of researchers, both to clarify the specific interaction of other amines in histamine metabolism and to elucidate the potential mechanisms of the so-called histamine-releasing foods.

## Figures and Tables

**Table 1:** Biogenic amines occurrence (mg/kg or L) found in different food and beverages from the Spanish market. Data are presented as average (standard deviation), P95 and minimum–maximum [11,29].

Foods	*n*	Occurrence of Biogenic Amines (mg/kg or L)					
		Histamine	Putrescine	Cadaverine	Tyramine	Spermidine	Spermine
Dry-fermented	424	21.57 (52.10)	68.23 (101.40)	32.45 (72.96)	140.9 (119.59)	5.39 (5.94)	25.12 (23.85)
sausages		129.95	280.3	172.10	378.51	18.80	59.94
		ND-474.82	ND-537.05	ND-658.05	ND-742.60	3.04–32.65	0.34–224.15
Cured, semi-cured	80	33.10 (77.10)	68.90 (141.30)	87.25 (283.55)	128 (264.41)	8.49 (12.40)	1.84 (4.46)
and		203.30	423.00	356.52	613.46	36.38	12.58
grated cheese		ND-389.86	ND-666.92	ND-2036.90	ND-1567.50	ND-68.92	ND-21.03
Soy-fermented	21	73.95 (184.51)	13.48 (6.71)	6.88 (11.97)	187.24 (446.59)	34.84 (38.23)	4.81 (5.24)
products		486.31	22.2	35.08	930	105.47	11.15
		ND-730.06	2.73–31.06	ND-36.95	ND-1730.17	ND-124.03	ND-21.89
Sauerkraut	5	43.74 (51.45)	232.66 (148.53)	76.49 (73.85)	43.47 (28.05)	5.29 (3.86)	0.85 (0.40)
		76.48	327.18	123.49	61.32	7.75	1.10
		7.36–80.12	127.63–337.68	24.27–128.71	23.63–63.3	2.56–8.02	0.56–1.13
Beer	176	1.23 (2.47)	3.16 (2.89)	1.28 (3.94)	6.31 (8.04)	0.48 (0.81)	0.19 (0.61)
		3.28	7.61	4.66	25.44	1.7	1.14
		ND-21.60	ND-14.50	ND-31.40	0.55–46.80	ND-6.30	ND-3.90
Wine	299	3.63 (5.86)	ND	ND	2.42 (2.47)	ND	ND
		12.3			7.5		
		0.09–34.25			ND-15.85		
Fresh white fish	31	1.14 (6.46)	1.33 (2.67)	1.61 (6.01)	1.03 (3.39)	2.25 (1.91)	6.78 (2.65)
		ND	7.43	4.8	6.51	5.32	11.27
		ND-36.55	ND-10.50	ND-33.65	ND-17.10	ND-7.85	2.05–13.50
Fresh oily fish	49	3.27 (15.71)	2.37 (6.71)	13.22 (67.40)	1.18 (5.43)	6.69 (3.09)	14.47 (9.75)
		6.66	5.02	10.45	2.19	11.39	31.67
		ND-111.26	ND-39.89	ND-400.23	ND-37.20	1.20–11.90	1.05 -37.03
Preserved and	151	10.03 (53.32)	2.79 (3.81)	7.41 (10.79)	8.23 (14.87)	3.61 (2.78)	7.48 (6.05)
semi-preserved		20.39	9.02	29.23	40.6	7.94	17.05
fish		ND-657.05	ND-21.15	ND-55.80	ND-88.50	0.37–11.80	ND-35.20
Seafood	7	ND	3.02 (3.01)	ND	0.15 (0.27)	4.03 (3.23)	10.63 (6.92)
			7.62		0.58	7.94	19.21
			1.44–9.79		ND-0.65	0.82–8.37	4.93–19.73
Fresh meat	199	ND	1.35 (2.66)	5.02 (14.55)	4.32 (8.62)	1.16 (4.59)	17.08 (4.59)
			3.04	28.71	35.89	3.4	29.55
			ND-9.68	ND-51.16	ND-38.77	ND-13.96	9.70–25.69
Cured meat	23	4.89 (22.70)	4.65 (5.18)	38.03 (92.82)	3.43 (10.56)	6.05 (0.92)	37.82 (10.46)
		3.54	9.24	49.64	42.58	6.88	42.58
		ND-150	ND-17.40	ND-305	ND-46.50	4.5–7.30	24.9–62.10
Milk	5	ND	ND	ND	ND	ND	ND
Yogurt	5	ND	2.04 (2.01)	ND	ND	1.07 (0.75)	0.28 (0.39)
			2.23			0.69	0.3
			ND-4.05			0.50–1.75	ND-0.50
Eggs	14	ND	ND	ND	ND	3.61 (1.54)	4.48 (1.72)
						4.43	5.27
						ND-4.47	0.32–5.31
Soybeans	5	ND	19.07 (4.39)	9.04 (1.09)	ND	99.55 (3.52)	25.92 (7.34)
			21.86	9.74		101.79	30.58
			15.96–22.17	8.27–9.81		97.06–102.04	20.73–31.10
Eggplant	23	39.42 (30.66)	34.30 (6.98)	ND	0.60 (0.90)	5.06 (1.93)	0.47 (0.48)
		98.84	46.29		2.24	7.7	1.29
		4.17–100.64	24.10–48.63		ND-2.27	2.54–7.97	ND-1.38
Spinach	18	31.77 (17.02)	4.48 (2.46)	ND (0.02)	2.05 (0.83)	28.22 (9.72)	3.33 (1.89)
		63.37	7.70	0.01	3.10	44.53	6.13
		9.46–69.72	0.14–9.20	ND-0.08	0.79–4.28	15.63–52.98	ND-8.85
Tomato	53	2.51 (4.08)	16.48 (6.93)	0.50 (0.48)	0.49 (0.92)	3.04 (1.41)	0.08 (0.16)
		13.83	30.16	1.42	1.21	5.69	0.36
		ND-17.07	6.29–35.55	ND-2.33	ND-6.38	2.91–7.90	ND-0.73
Pumpkin	13	ND	9.87 (6.19)	0.58 (0.78)	ND	10.32 (2.83)	1.77 (1.99)
			19.17	1.82		13.88	5.21
			2.95–24.23	ND-2.15		6.19–14.98	0.5–6.88
Apricot	4	ND	ND	ND	ND	5.86 (1.59)	ND
						6.50	
						4.16–7.68	
Avocado	5	ND	ND	ND	1.81 (2.06)	3.15 (3.27)	4.50 (2.52)
					4.65	6.69	7.61
					0.58–5.44	0.18–6.72	2.02–7.92
Banana	8	ND	37.94 (8.32)	ND	0.53 (0.79)	11.91 (2.90)	1.33 (0.97)
			47.37		ND	15.10	2.67
			25.50–49.49		ND-1.85	7.62–15.79	ND- 2.75
Citrus fruits	38	ND	79.75 (44.36)	ND	ND	2.57 (1.28)	0.12 (0.36)
			146.16			4.86	1.02
			1.21–173.81			0.18–6.24	ND-1.14
Cherry	5	ND	3.42 (0.06)	ND	ND	2.37 (0.16)	ND
			3.46			2.47	
			3.42–3.46			2.26–2.47	
Grapes	10	ND	2.69 (0.34)	ND	ND	5.25 (2.61)	2.59 (0.11)
			4.05			8.6	2.56
			1–4.30			ND-9.70	2.35–2.68
Kiwi	13	ND	1.47 (0.47)	ND	ND	5.35 (1.06)	0.73 (0.56)
			2.07			6.39	1.41
			0.48–2.17			2.72–6.39	ND-1.50
Papaya	6	ND	7.25 (5.80)	ND	ND	14.35 (4.32)	1.16 (1.59)
			11.86			15.45	2.06
			ND-12.48			10.32–19.07	ND-2.99
Pineapple	5	ND	2.69 (1.42)	ND	ND	1.92 (1.26)	0.48 (0.21)
			3.89			3.15	0.75
			0.56–3.97			0.27–3.18	0.32–0.77
Plum	6	ND	ND	ND	4.02 (4.32)	2.68 (0.30)	1.74 (2.47)
					6.76	2.87	3.31
					0.96–7.07	2.47–2.89	ND- 3.48
Red fruits	7	ND	ND	ND	7.37 (1.03)	5.58 (1.16)	1.97 (1.61)
					9.36	2.54	1.65
					3.34–11.52	0.78–3.98	ND–3.73
Strawberries	9	ND	3.77 (1.52)	ND	ND	6.00 (1.56)	0.46 (0.69)
			6.09			8.52	1.5
			2.04–6.41			4.62–9.86	ND-1.62
Olives	5	ND	2.64 (1.58)	ND	1.95 (1.85)	ND	ND
			4.2		3.7		
			1.54–4.45		0.28–3.94		
Nuts	47	ND	4.40 (7.11)	0.25 (1.69)	0.11 (0.41)	28.64 (24)	11.14 (8.91)
			12.58	ND	0.66	55.23	23.73
			ND-39.51	ND-11.58	ND-2.63	6.21–140.55	ND-50.81
Chocolate	15	ND	0.41 (0.65)	0.42 (0.96)	3.70 (1.24)	3.11 (0.70)	2.00 (0.90)
			1.89	ND	5.69	4.23	2.65
			ND- 1.98	ND-2.78	2.27–5.81	2.17–4.65	ND-2.72
Tea	9	ND	2.61 (0.49)	ND	5.07 (3.80)	5.86 (1.18)	18.32 (5.31)
			3.12		8.34	6.59	22.84
			2.66–3.37		ND-10.08	3.66–7.64	8.23–23.94
